# Activation of ATF4 triggers trabecular meshwork cell dysfunction and apoptosis in POAG

**DOI:** 10.18632/aging.202677

**Published:** 2021-03-10

**Authors:** Ying Ying, Ran Xue, Yangfan Yang, Sarah X Zhang, Hui Xiao, Huazhang Zhu, Jingming Li, Guo Chen, Yiming Ye, Minbin Yu, Xing Liu, Yimin Zhong

**Affiliations:** 1 State Key Laboratory of Ophthalmology, Zhongshan Ophthalmic Center, Sun Yat-Sen University, Guangzhou, Guangdong, China; 2 Department of Physiology, Shenzhen University Health Science Center, Shenzhen University, Shenzhen, Guangdong, China; 3 Department of Ophthalmology, The First Affiliated Hospital of Xi’an Jiaotong University College of Medicine, Xi an, Shanxi, China; 4 Department of Ophthalmology and Ross Eye Institute, University at Buffalo, State University of New York, Buffalo, NY 14209, USA; 5 SUNY Eye Institute, State University of New York, New York, NY 10036, USA; 6 Department of Biochemistry, University at Buffalo, State University of New York, Buffalo, NY 14203, USA

**Keywords:** activating transcription factor 4, trabecular meshwork, primary open angle glaucoma, oxidative stress, endoplasmic reticulum stress

## Abstract

Primary open angle glaucoma (POAG) is the leading cause of irreversible blindness. Dysfunction of the trabecular meshwork (TM), resulting in decreased outflow of aqueous humor and increased intraocular pressure (IOP), plays an important role in the pathogenesis of POAG. However, the underlying mechanisms still remain unclear. In this study, we demonstrated that the eIF2-α/ATF4/CHOP branch of unfolded protein response (UPR) was activated in human trabecular meshwork cells (HTMCs) upon tert-butyl hydroperoxide (TBHP) exposure. Inhibition of ATF4 ameliorated TBHP-induced apoptosis and inflammatory cytokine production, while ectopic expression of ATF4 increased the expression of endothelial leukocyte adhesion molecule (ELAM)-1 and IL-8 in HTMCs. Furthermore, we found that ATF4 inhibition reduced tunicamycin-induced caspase-3 activation, ROS production, ELAM-1 expression, and HTMCs phagocytosis impairment. By an *in vivo* study in mice, we showed that overexpression of ATF4 in the TM induced C/EBP homologous protein (CHOP) expression and TM cells apoptosis, contributing to inflammatory cytokine production, and probably IOP elevation. More importantly, upregulation of ATF4 and CHOP, and colocalization of ATF4 with ELAM-1 were found in the TM of POAG patients. These results suggest that ATF4 is a critical mediator of oxidative stress and ER stress-induced TM cell dysfunction and apoptosis in POAG.

## INTRODUCTION

Glaucoma has long been recognized as the leading cause of irreversible blindness. Primary open angle glaucoma (POAG) is one of the most common forms of glaucoma [1, 2]. The pathogenesis of POAG is not yet fully understood. But it is considered a progressive optic neuropathy often caused by elevated intraocular pressure (IOP). Abnormally increased resistance to aqueous humor outflow caused by dysfunction of the trabecular meshwork (TM) may play an important role in the pathogenesis of POAG [1, 3].

The molecular pathways that lead to dysfunction of TM are not well understood but oxidative stress, which is a significant risk factor, is probably involved. TM cells submitted to oxidative stress exhibit POAG-typical changes such as cell death [4–6], extracellular matrix accumulation [7, 8], and release of inflammatory markers [8–10]. Furthermore, oxidative stress may destroy the redox environment of the endoplasmic reticulum (ER), thus causing decreased protein folding and ER stress [11]. Growing evidence suggests that ER stress-triggered signaling pathways, such as unfolded protein response (UPR), regulate cell energy metabolism, redox status, inflammation, and cell survival. But the cross-talk between oxidative stress and ER stress in the TM cells dysfunction remains largely unknown.

Activating transcription factor 4 (ATF4) is a major molecular component of the UPR signaling pathway. Activation of ATF4 induces the expression of C/EBP homologous protein (CHOP), a key mediator of ER stress-associated apoptosis [12–16]. Moreover, ATF4 also functions as an important regulator of inflammatory genes [17, 18]. Previously, we demonstrated that ATF4 was required for diabetes-induced expression of ICAM (intercellular adhesion molecule)-1 and vascular endothelial growth factor (VEGF) in retinal Müller cells and plays a critical role in retinal inflammation in diabetic retinopathy [19]. However, the role of ATF4 in dysfunction and apoptosis of TM cells is yet to be determined.

In the current study, we investigated the potential role of ATF4 in oxidative stress and ER stress-induced TM cells dysfunction and apoptosis. Our results suggest that ATF4 is an important pathogenic factor in POAG through regulation of inflammatory gene expression, phagocytosis and apoptosis in TM cells.

## RESULTS

### TBHP activates ER stress, upregulates the expression of ATF4, and induces apoptosis in Human Trabecular Meshwork Cells (HTMCs)

**Figure 1:**
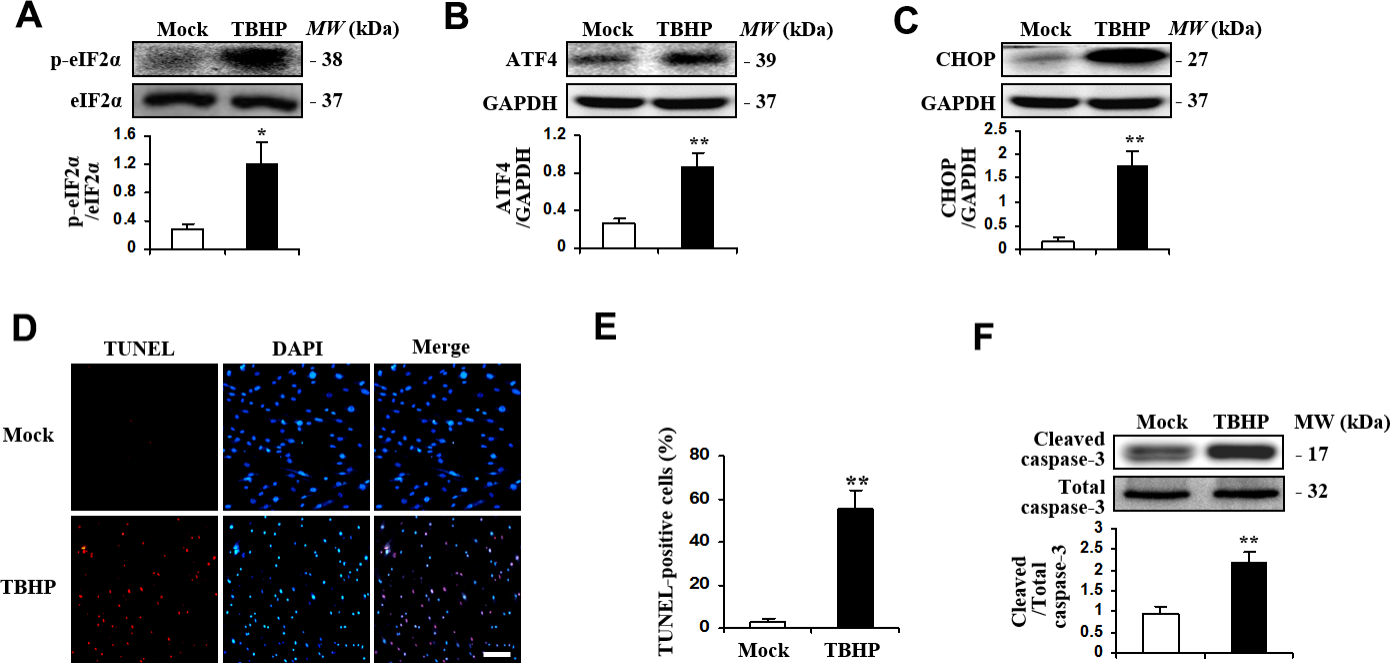
**TBHP activated the expression of transcription factor ATF4 and induced apoptosis in HTMCs.** HTMCs were exposed to media in the absence (Mock) or presence of 50 μM of TBHP for 12 h. (**A**–**C**) Western blot analysis for phospho-eIF2α, total eIF2α, ATF4, CHOP and GAPDH. Intensities were quantified and normalized against the level of total proteins (eIF2α) or GAPDH (mean ± SEM, n = 3). (**D**) Representative images of immunostaining for apoptotic (TUNEL-positive, red) HTMCs. Nuclei were labeled with DAPI (blue). Scale bar represents 100 μm. (**E**) Quantification of apoptotic nuclei by Image-Pro Plus software. Values are expressed as the percentage of TUNEL-positive cells to total (DAPI-positive) cells (mean ± SEM, n = 3). (**F**) Western blot analysis for cleaved caspase-3. Intensities were quantified and normalized against the level of total caspase-3 (mean ± SEM, n = 3). ^*^P<0.05 and ^**^P <0.01 vs. Mock.

We firstly examined if TBHP was sufficient to induce ER stress and ATF4 activation in HTMCs. HTMCs were exposed to culture media with or without TBHP (50 μM) for 12 h, ER stress markers including phosphorylation of eIF2-α (eukaryotic translation initiation factor 2-α), expression of ATF4 and its major target gene, CHOP, were measured by Western blot analysis. The results show that TBHP treatment resulted in increased phosphorylation of eIF2-α (Figure 1A). In addition, ATF4 and CHOP expression was significantly increased (Figure 1B, 1C). We further detected apoptosis of HTMCs induced by TBHP. We found markedly increased TUNEL staining in HTMCs after being exposed to TBHP for 12 h (Figure 1D, 1E). Caspase-3 activation, indicated by expression of cleaved caspase-3 (Figure 1F), was also observed in HTMCs after TBHP treatment.

### ATF4 is required for oxidative stress-induced apoptosis in HTMCs

**Figure 2:**
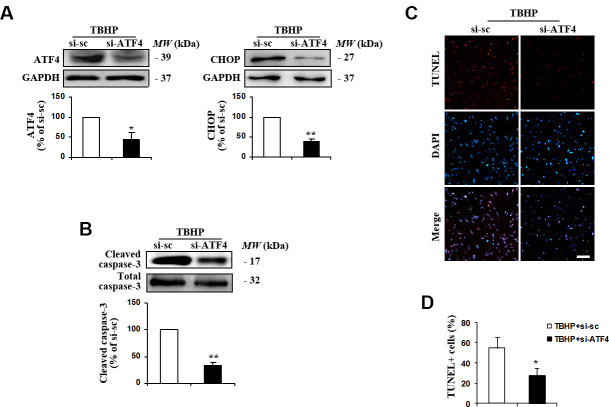
**Suppression of ATF4 expression by siRNA transfection inhibited TBHP-induced apoptosis of HTMCs.** HTMCs were transfected with siRNA specific to ATF4 (si-ATF4) or to scramble sequences (si-sc) for 48 h, after which they were exposed to 50 μM of TBHP for additional 12 h. (**A**) The suppression efficiency of ATF4 using si-RNA transfection and the expression of its downstream target CHOP were determined by Western blot analysis (mean ± SEM, n = 3). (**B**) Levels of cleaved caspase-3 were examined by Western blot. Intensities of protein expression were quantified, normalized against the level of total caspase-3 and expressed as relative changes to protein abundance in cells transfected with scramble control (si-sc) (mean ± SEM, n = 3). (**C**) Apoptotic cells were examined by TUNEL staining (red, TUNEL; blue, DAPI; scale bar, 100 μm). (**D**) Numbers of apoptotic cells were quantified and expressed as the percentage of TUNEL-positive to DAPI-positive cells (mean ± SEM, n = 3). ^*^P<0.05 and ^**^P<0.01 vs. TBHP+si-sc. TBHP+si-sc, exposure of HTMCs transfected with si-sc to TBHP. TBHP+si-ATF4, exposure of HTMCs transfected with si-ATF4 to TBHP.

ATF4 has been shown to induce stress response genes involved in oxidative stress and to upregulate pro-apoptotic transcription factors in neurons [20]. To assess the potential role of ATF4 in TBHP-induced HTMCs apoptosis, we manipulated ATF4 expression using ATF4 small interfering RNA (siRNA). We found that downregulation of ATF4 (Figure 2A) markedly reduced the expression of its downstream target CHOP (Figure 2A) and alleviated oxidative stress-induced apoptosis, indicated by decreases in cleaved caspase-3 expression (Figure 2B) and the number of TUNEL-positive cells (Figure 2C, 2D). These data suggest that TBHP induces apoptosis of HTMCs through ATF4.

### Activation of ATF4 causes inflammatory gene expression in HTMCs

**Figure 3:**
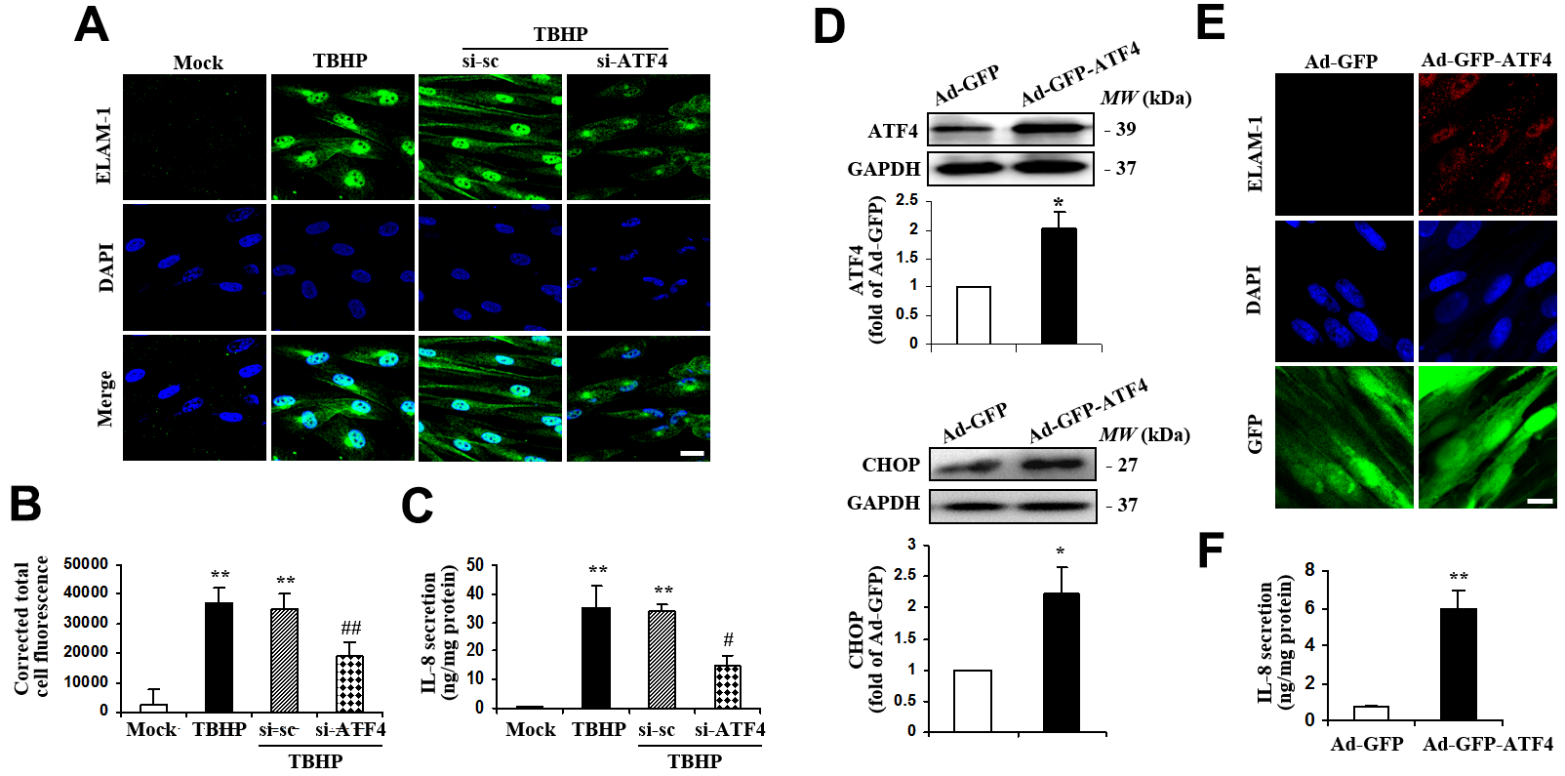
**ATF4 is an important mediator of ELAM-1 expression and IL-8 secretion in HTMCs.** (**A**–**C**) HTMCs were exposed to media in the absence (Mock) or presence of 50 μM of TBHP for 12 h. Alternatively, cells were transfected with si-ATF4 or si-sc for 48 h, followed by incubation with 50 μM of TBHP for additional 12 h. (**A**) Representative images of subcellular expression of ELAM-1 as examined by indirect immunofluorescence (green, ELAM-1. blue, DAPI. scale bar, 40 μm). (**B**) Levels of cellular immunofluorescence of ELAM-1 were quantified and expressed as corrected total cell fluorescence (mean ± SEM, n = 3). (**C**) IL-8 secretion was assayed in culture supernatant of HTMCs using an IL-8 ELISA kit. Values were normalized for total protein at the respective treatment (mean ± SEM, n = 3). ^**^P <0.01 vs. Mock, ^#^*P* <0.05 and ^##^*P* <0.01 vs. TBHP+si-sc. TBHP+si-sc, exposure of cells transfected with si-sc to TBHP. TBHP+si-ATF4, exposure of cells transfected with si-ATF4 to TBHP. (**D**–**F**) A recombinant adenovirus coding ATF4 (Ad-GFP-ATF4) was constructed to express GFP protein as a marker for the identification of infected cells. Cultured HTMCs were infected with Ad-GFP-ATF4 or empty vector (Ad-GFP) for 72 h. (**D**) Western blot analysis for ATF4 and CHOP. Intensities of protein expression were quantified, normalized against the level of GAPDH and expressed as relative changes to Ad-GFP (mean ± SEM, n = 3). (**E**) Subcellular expression of ELAM-1 was detected by immunofluorescent staining (red, ELAM-1. blue, DAPI. green, GFP. scale bar, 20 μm). (**F**) IL-8 secretion was assayed using ELISA. Values were normalized for total protein at the respective treatment (mean ± SEM, n = 3). ^*^P <0.05, ^**^P <0.01 vs. Ad-GFP.

Previous studies suggest that upregulation of inflammation-associated genes might be involved in the progression of glaucoma [21–24], so we next determined the expression of IL-8 and endothelial leukocyte adhesion molecule (ELAM)-1 in HTMCs exposed to TBHP. We found that TBHP remarkably increased ELAM-1 expression (Figure 3A, 3B) and IL-8 secretion (Figure 3C) in HTMCs. Knockdown of ATF4, which has previously been shown to be a major transcription factor required for inflammatory gene expression in other cell types [17, 18, 25, 26], significantly attenuated ELAM-1 (Figure 3A, 3B) and IL-8 expression (Figure 3C) in TBHP-challenged HTMCs. Moreover, ectopic expression of ATF4 (Figure 3D) upregulated the protein level of ELAM-1 (Figure 3E) and secretion of IL-8 (Figure 3F). These results strongly suggest a critical role of ATF4 in mediating TBHP-induced inflammatory factor production in HTMCs.

### ATF4 mediates ER stress-induced ROS production, apoptosis, and phagocytotic dysfunction in HTMCs

**Figure 4:**
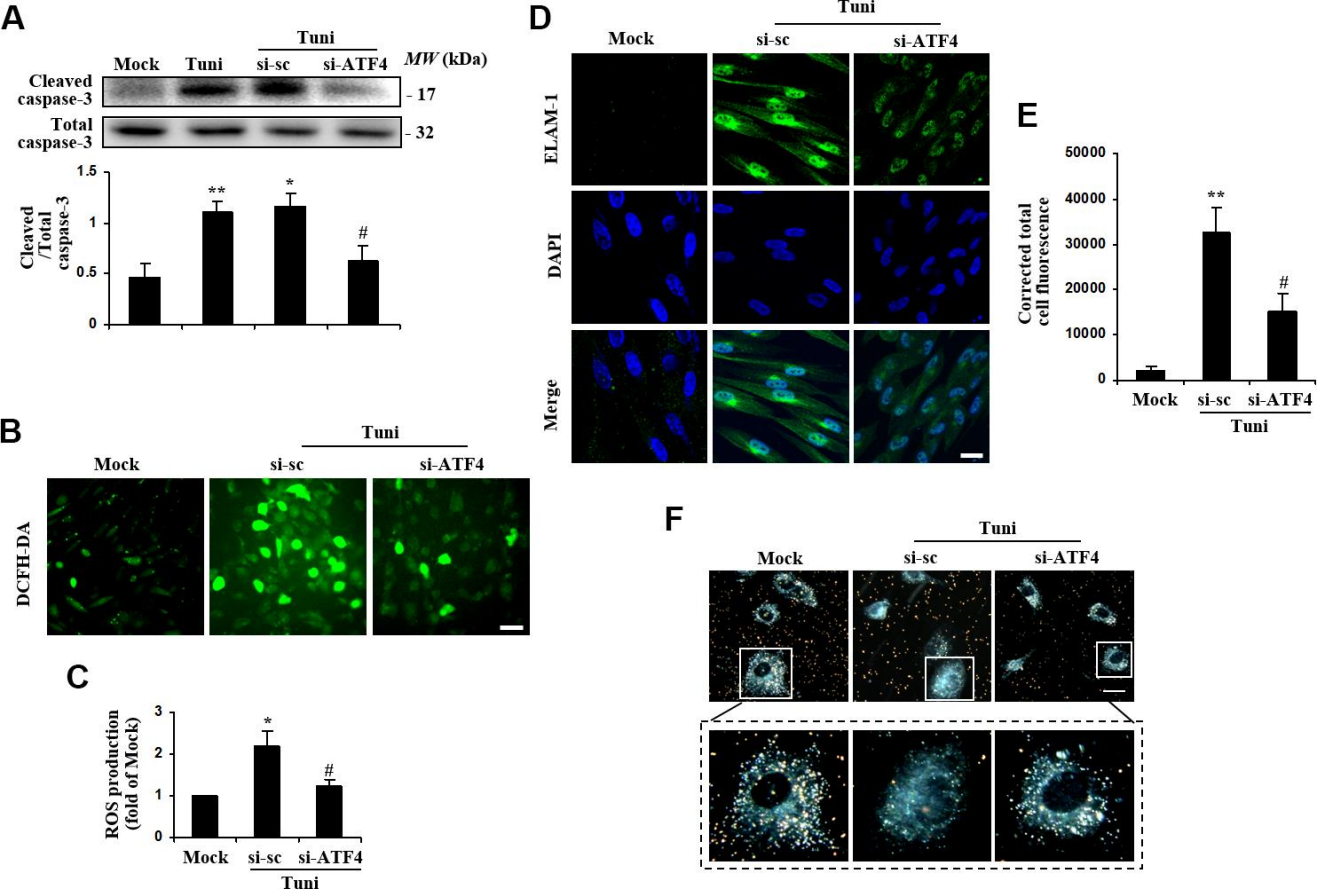
**Suppression of ATF4 prevented tunicamycin-induced apoptosis and ROS generation, and rescued the phagocytotic activity of HTMC.** HTMCs were exposed to media in the absence (Mock) or presence of 100 ng/ml of tunicamycin (Tuni) for 24 h. Alternatively, cells were transfected with si-ATF4 or si-sc for 48 h, followed by incubation with 100 ng/ml of tunicamycin (Tuni) for additional 24 h. (**A**) Expression of cleaved caspase-3 was determined by Western blot analysis and quantified by densitometry (mean ± SEM, n = 3). (**B**) Representative images of ROS production in cells incubated with DCFH-DA (green. scale bar, 60 μm). (**C**) Quantification of intracellular ROS production. Values are expressed as the fold increase from Mock in ROS content evaluated by fluorescence intensity (mean ± SEM, n = 3). (**D**) Representative images of subcellular expression of ELAM-1 by indirect immunofluorescence (green, ELAM-1. blue, DAPI. scale bar, 40 μm). (**E**) Levels of cellular immunofluorescence of ELAM-1 were measured and expressed as corrected total cell fluorescence (mean ± SEM, n = 3). (**F**) Phagocytosis of colloidal gold by HTMCs was examined by dark field microscope (gold, colloidal gold). Representative images were mounted in upper panels (scale bar, 20 μm). Lower panels show magnified images of individual cells. ^*^P<0.05 vs. Mock, ^**^P <0.01 vs. Mock, ^#^*P*<0.05 vs. Tuni+si-sc.

Recent studies suggest that ER stress-triggered signaling pathways regulate the status of oxidative stress and cell survival [27, 28]. To assess if ATF4 regulates oxidative stress and cell survival in HTMCs, we transduced the cells with siRNA to downregulate ATF4 expression and then exposed the cells to tunicamycin to induce ER stress. The results demonstrated that blockade of ATF4 drastically attenuated tunicamycin-induced caspase-3 activation (Figure 4A). Furthermore, ROS level was reduced markedly in ATF4-deficient cells exposed to tunicamycin (Figure 4B, 4C). This indicates that ATF4 is an important mediator of oxidative stress and apoptosis induced by ER dysfunction in HTMCs. Loss of ATF4 also remarkably alleviated tunicamycin-induced ELAM-1 expression (Figure 4D).

Phagocytosis is an important function for HTMCs related to extracellular matrix remodeling and regulation of IOP [29, 30]. As shown in Figure 4E, HTMCs under normal conditions were highly phagocytic as indicated by their ability to uptake colloidal gold particles. Notably, the phagocytic activity of tunicamycin-stressed HTMCs was markedly impaired, as demonstrated by reduced number of colloidal gold particles within the cells. Knockdown of ATF4 restored the phagocytic activity of HTMCs, suggesting that ATF4 is essential for ER stress-induced HTMC phagocytotic dysfunction.

### Activation of ATF4 induces TM cell apoptosis contributing to elevation of IOP in mice

**Figure 5:**
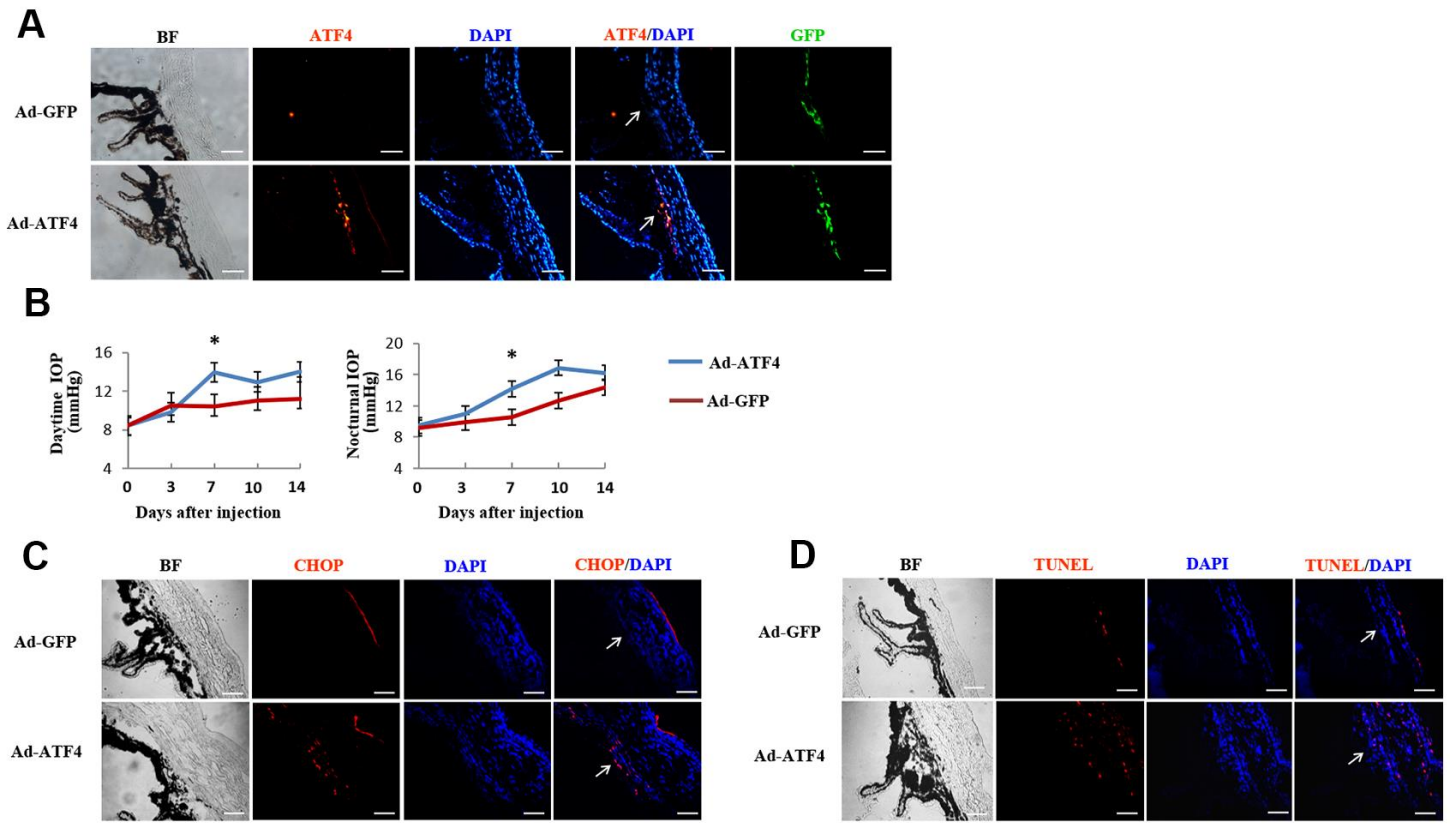
**Activation of ATF4 in mice TM induces apoptosis and increase of intraocular pressure.** ATF4 adenoviral vectors or GFP adenoviral vectors as control were injected into the anterior chamber of 6~8 weeks old C57BL/6J mice. (**A**) Visualization of the GFP fluorescence (green) of adenovirus in mice TM after anterior chamber injection for 24 h and increased ATF4 fluorescence (red) could be seen in the ATF4 adenovirus injected eye (Ad-ATF4) compared to the control (Ad-GFP). (**B**) Intraocular pressure (IOP) of the mice was measured day (left) and night (right) before and after anterior chamber injection. IOP was markedly elevated in the Ad-ATF4 group (n = 8) compared to the control (Ad-GFP) (n = 7) 7 days after the injection, *P<0.05 vs. Ad-GFP. (**C**) Immunofluorescent staining of the iridocorneal angle showing increased expression of CHOP (red) in the TM of Ad-ATF4 group after anterior chamber injection for 7 days (n = 3). (**D**) TUNEL staining of the iridocorneal angle 7 days after anterior chamber injection (n = 3). Arrows mark the TM. Blue, nuclear staining with DAPI. BF, bright field. TM, trabecular meshwork. Scale bar, 50 μm.

To determine the effect of ATF4 on TM cell dysfunction *in vivo*, we overexpressed ATF4 in TM by injecting Ad-ATF4 into the anterior chamber of adult C57BL/6 mice. Ad-GFP was used as a control. Green fluorescence, which indicates successful viral transduction, was visualized 24 hours after injection and increased expression of ATF4 was detected in the TM of Ad-ATF4-treated eyes (Figure 5A). IOP was measured daily before and within 14 days after the injection. Markedly elevated IOP was found 7 days after the injection in Ad-ATF4-treated eyes compared to Ad-GFP controls (Figure 5B). Meanwhile, increased expression of CHOP was observed in the TM of Ad-ATF4-treated eyes (Figure 5C). Increased number of TUNEL positive cells was also detected in the Ad-ATF4 group (Figure 5D). These data indicate that activation of ATF4 is sufficient to induce TM cell apoptosis, and probably increased IOP in mice.

### Activation of ATF4 stimulates inflammatory cytokines production in iridocorneal angle

**Figure 6:**
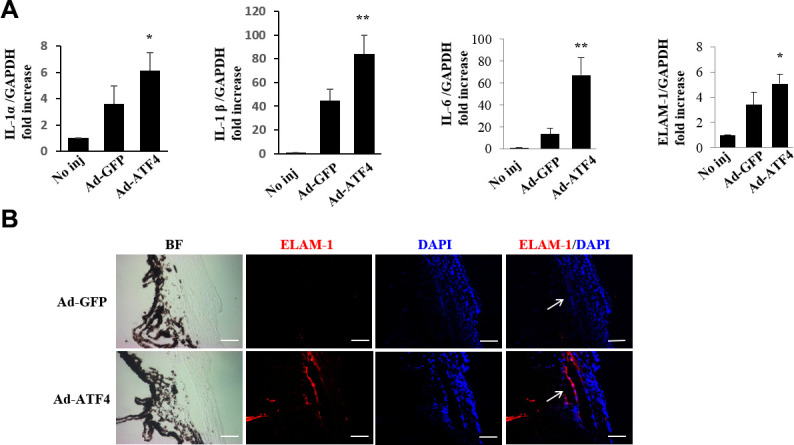
**Activation of ATF4 mediates inflammatory cytokines expression in mice TM.** (**A**) mRNA expression of inflammatory cytokines, IL-1α, IL-1β, IL-6 and ELAM-1 in the iridocorneal angle tissues of Ad-ATF4, Ad-GFP and no-injection group (No inj) was determined by real-time RT-PCR 3 days after injection (mean ± SEM, n = 4). ** *P*<0.01 vs. Ad-GFP, * *P*<0.05 vs. Ad-GFP. (**B**) Increased expression of ELAM-1 (red) was detected in the TM of the Ad-ATF4 group 7 days after anterior chamber injection (n = 3). Arrows mark the TM. Blue, nuclear staining with DAPI. BF, bright field. Scale bar, 50 μm.

We next examined the effects of ATF4 on inflammatory cytokine expression in mouse TM. We found that the mRNA levels of IL-1α, IL-1β, IL-6 and ELAM-1 in the iridocorneal angle tissues were significantly increased in the Ad-ATF4-treated eyes 3 days post-injection (Figure 6A). Increased immunofluorescent staining of ELAM-1 was observed in the Ad-ATF4-treated eyes 7 days after injection (Figure 6B). These results suggest that upregulation of ATF4 increases the production of inflammatory cytokines in the iridocorneal angle, which could even precede cell apoptosis in TM.

### Upregulation of ATF4, ELAM-1 and CHOP in TM tissues of POAG patients

**Figure 7:**
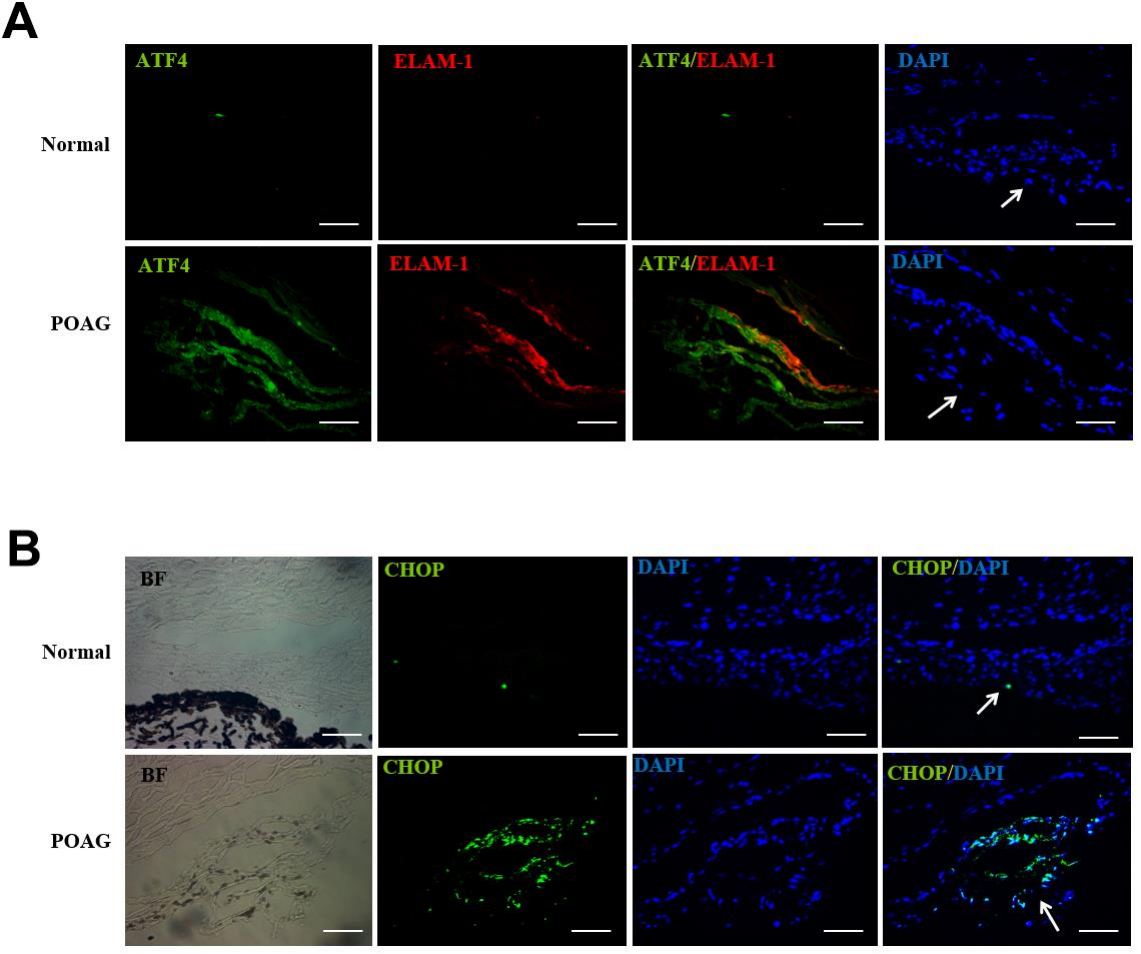
**Up-regulation of ATF4, ELAM-1 and CHOP in the TM tissues of POAG patients.** (**A**) Frozen sections of human TM tissues of POAG patients (n = 3) or normal controls (n = 4) were double stained with ATF4 (green) and ELAM-1 (red) antibodies. Note the partial colocalization of immunofluorescence in the TM of glaucomatous eyes. (**B**) Immunohistochemistry showing increased CHOP (green) expression in the TM of POAG patients. Arrows mark the TM. Blue: nuclear staining with DAPI. BF, bright field. Scale bar, 50 μm Supplementary Figure 2.

Finally, we examined the expression of ATF4, CHOP, and ELAM-1 in TM sections from POAG patients. We found that the expression of ATF4, ELAM-1 (Figure 7A) and CHOP (Figure 7B) was significantly increased in the TM of glaucomatous eyes. Furthermore, we observed colocalization of ATF4 with ELAM-1, which is a glaucoma marker (Figure 7A). These findings suggest a potential role of ATF4 in the pathogenesis of glaucoma [22].

## DISCUSSION

The TM is the primary target tissue of glaucoma. Cumulative oxidative damage and dysfunction of TM cells are believed to contribute to the development and progression of the disease [9, 31, 32]. However, the molecular mechanisms underlying oxidative damage of TM cells are poorly understood. In the current study, we demonstrate that TBHP-induced ATF4 activation plays an important role in this process. We show that the UPR branch of eIF2α/ATF4/CHOP is activated by TBHP and activation of ATF4 is required for oxidative stress-induced apoptosis, inflammatory cytokine production, and phagocytotic dysfunction of TM cells.

Our study confirmed a role of ATF4 in TM cells apoptosis induced by oxidative and ER stress. Previous studies have shown that ATF4 functions as a transcriptional activator that propagates death response to oxidative stress in the nervous system [20]. ATF4 was also found to play a key role in sorafenib-induced renal cell carcinoma apoptosis [33]. Our results support these findings and demonstrate that ATF4 mediates TM cells apoptosis both *in vitro* and *in vivo*. Inhibition of ATF4 attenuated TBHP-induced caspase-3 expression and TUNEL staining in HTMCs. Activation of ATF4 increased TUNEL staining in mice TM. These effects are probably mediated by CHOP, a central player in ER stress-induced apoptosis [13, 14, 34, 35]. We found that knockdown or overexpression of ATF4 significantly alters the expression of CHOP in HTMCs, and CHOP was upregulated simultaneously with ATF4-induced TUNEL staining in mice TM. The exact role of CHOP in TM cell apoptosis and cell death related to glaucoma warrants future investigation.

Furthermore, our results indicate that ATF4 is an important mediator of ELAM-1 expression in TM. ELAM-1 is a disease marker for glaucomatous TM [22, 24]. It is activated by IL-1 and NF-κB and consistently present in glaucomatous tissue specimens and TM-cells lines [22]. We found that inhibiting ATF4 attenuates TBHP and tunicamycin-induced ELAM-1 expression, and, conversely, overexpression of ATF4 is sufficient to induce ELAM-1 accumulation and IL-8 secretion in HTMCs. ATF4 regulation of ELAM-1 expression was further confirmed by an *in vivo* study. Induction of ATF4 in TM played an important role in increased expression of ELAM-1, IL-1α, IL-1β, and IL-6, and probably in inducing glaucoma phenotype (elevated IOP) in mice. In addition, we found that ATF4 colocalized with ELAM-1 in the TM tissue of POAG patients. These results strongly suggest a role of ATF4 in ELAM-1 upregulation in glaucoma TM. Further studies are needed to elucidate how ATF4 regulates ELAM-1 expression in TM cells.

Another important finding from our study is that ATF4 is implicated in the regulation of phagocytosis, which is a pivotal function of HTMCs in maintaining the normal outflow pathway of the eye. Recent studies have shown that ER stress, due to compromised ability of the UPR to eliminate misfolded mutant or damaged proteins, including myocilin, results in functional impairment and apoptosis of TM cells [36–39]. Herein, we demonstrated that induction of ER stress by tunicamycin decreased phagocytosis in HTMCs. Impaired phagocytosis is associated with increased deposition of extracellular matrix material in the outflow pathway and elevated IOP [29, 30]. Inhibition of ATF4 rescued ER stress-induced impaired phagocytosis in HTMCs. We also found that overexpression of ATF4 impaired the phagocytotic activity of HTMCs (Supplementary Figure 1). These data suggest that ATF4 upregulation contributes to HTMCs dysfunction in glaucoma. The mechanisms underpinning ATF4-mediated HTMCs phagocytic impairment are yet to be determined.

In summary, our study suggests that ATF4 is a critical mediator of oxidative stress and ER stress-induced TM cell dysfunction and apoptosis. Targeting ATF4 may provide a novel and promising approach to improve TM function and prevent TM cell loss in POAG.

## MATERIALS AND METHODS

### Materials

Tunicamycin and TBHP were obtained from Sigma-Aldrich (St. Louis, MO, USA). Protease and phosphatase inhibitors were purchased from Selleckchem (Houston, TX, USA). Biotinylated secondary antibody and fluorescein isothiocyanate avidin were purchased from Invitrogen (Carlsbad, CA, USA). DAPI (4’, 6-diamidino-2-phenylindole) was obtained from Vector Laboratories (Burlingame, CA, USA).

### Human TM specimens

Three TM samples were obtained from three POAG patients undergoing trabeculectomy. Patients with interfering ocular pathology or systemic diseases were excluded. All patients had received anti-glaucomatous eye drops for no more than one month. Four normal control cadaver eyes were obtained from the eye bank of Zhongshan Ophthalmic Center (ZOC). All control TM tissues were obtained from donor eyes without a history of glaucoma or elevated IOP. Control TM specimens were dissected within 12 h of enucleation. This study adhered to the tenets of the Declaration of Helsinki. Ethical approval was given by the institutional review board of ZOC. Informed consent was obtained from each patient.

### Cell culture

Primary HTMCs isolated from the corneoscleral rim tissues of a 65-year-old donor were purchased from ScienCell Research Labs (Carlsbad, CA, USA). To identify the primary TM cells, the expression of known markers, Matrix Gla Protein (MGP) (#ab86233, Abcam, Cambridge, MA, USA), Chitinase-3-Like-1 (CH3L1) (#ab77528, Abcam), Dexamethasone-induced cross-linked actin networks (CLANs) (#FAK100; Millipore, Billerica, MA, USA), and water channel Aquaporin 1 (AQP1) (#ab65837, Abcam) were determined using immunofluorescence staining (Supplementary Figure 3). Cells were maintained in Trabecular Meshwork Cell Medium (TMCM) containing 2% fetal bovine serum (FBS), 1% trabecular meshwork cell growth supplement (TMCGS), and 1% Penicillin/Streptomycin. Cells within 6th passage were used. For tunicamycin or TBHP treatment, HTMCs at 80% confluence or after 24-48 h transfection with siRNA were incubated with the maintenance medium (TMCM with 0.5% FBS) in the presence of tunicamycin (100 ng/ml) or TBHP (50 μM) at 37° C for 48 h or 12 h, respectively. After incubation, medium was collected and cells were harvested for biochemical or immunocytochemical analysis.

### Animal studies

C57/BL6 mice were purchased from Guangdong Medical Laboratory Animal Center. Care, use and treatment of all animals were in strict agreement with the guidelines of the Association for Research in Vision and Ophthalmology Statement for the Use of Animals in Ophthalmic and Visual Research and approved by the institutional animal care and use committees in ZOC. The environment was kept at 20-25° C with a 12 h light/12 h dark cycle.

### IOP measurements

Mice were anesthetized using isoflurane plus oxygen. IOP was measured with a rebound tonometer (Icare, Helsinki, Finland). Daytime IOP was measured between 8 and 10 am. And nocturnal IOP was measured between 9 and 11 pm.

### Anterior chamber injection

The mice were anesthetized with 40 mg/ml chloral hydrate at a dose of 0.01 ml/g by intraperitoneal injection. A recombinant adenovirus coding for ATF4 with N-terminal GFP tag (Ad-ATF4) (3.6×10^8^ PFU/eye, Obio Technology Corp., Ltd, Shanghai, China) or an empty vector control (Ad-GFP) (4×10^8^PFU/eye) was injected in the anterior chamber at a volume of 2 μl into one eye of each animal. Mice that developed hyphema or lens opacities after injection were excluded from further study.

### Transfection of siRNA or transduction of recombinant adenovirus in HTMCs

HTMCs at 50% confluence were transfected with siRNA specific to ATF4 (si-ATF4) or scramble control (si-sc) (Santa Cruz Biotechnology, Santa Cruz, CA, USA) using Lipofectamine 3000 (Invitrogen) following the manufacturer’s instruction. Cells were maintained overnight with serum-free DMEM medium before treatment. HTMCs at 80%-90% confluence were transduced with Ad-ATF4 or control Ad-GFP, respectively, at a multiplicity of infection (MOI) of 50. Forty-eight hours after transduction, the adenovirus was removed and the cells were maintained overnight in serum-free DMEM medium without Penicillin/Streptomycin before the desired treatment.

### Immunohistochemistry

For frozen sections, TM tissues were fixed with 4% paraformaldehyde for 1 h and then dehydrated with a series of sucrose solutions (10–30%) before optimum cutting temperature embedding. Cross sections of the TM were obtained using a cryostat (Leica Biosystems, Buffalo Grove, IL, USA). Sections were immunostained using anti-ATF4 (1:100, #ab23760, Abcam), anti-CHOP (1:100, #sc-575, Santa Cruz Biotechnology), and anti-CD62E (1:500, #550290 from Pharmingen (San Diego, CA, USA) or #S9555 from Sigma-Aldrich) antibodies overnight at 4° C, followed by Cy3-conjugated secondary antibody from Invitrogen, or Alexa Fluor 568 from Thermo Fisher Scientific (Waltham, MA, USA), or followed by biotinylated secondary antibody and fluorescein isothiocyanate avidin. Nuclei were mounted with DAPI-mounting solution. Slides were visualized and photographed under a fluorescent microscope (Axioplan 2 imaging, Carl Zeiss, Heidenheim, Germany).

### Immunocytochemistry

HTMCs grown on glass coverslips were fixed with 4% paraformaldehyde and then permeabilized in 0.1% Triton X-100. After blocking with 1% BSA for 30 min, cells were incubated with primary antibody (anti-CD62E, 1:100, ab18981, Abcam) overnight at 4° C followed by Alexa-Fluor-488 (Invitrogen) labelled secondary antibody at 37° C for 1 h and then counterstained with DAPI. Fluorescence was observed and pictures were taken under a FluoView FV1000 confocal microscope (Olympus). The level of cellular fluorescence was determined using Image-Pro Plus software. The corrected total cell fluorescence (CTCF) was calculated using the formula: CTCF = Integrated Density – (Area of selected cell X Mean fluorescence of background readings). At least seventy-five cells were quantified for each treatment from three independent experiments.

### Western blot analysis

Cells were homogenized in hypotonic Tris buffer (10mM Tris, 0.2 mM MgCl_2_, PH 7.4) containing protease and phosphatase inhibitor cocktail using a probe sonicator, followed by centrifugation at 12,000 rpm for 15 min at 4° C. The resultant supernatant was used for the immunoblot analysis. Protein concentration was quantified using protein assay reagent (Solarbio, Beijing, China). Equal amounts of protein were resolved by SDS-PAGE gel and electrotransferred to nitrocellular membranes. After blocking, membranes were blotted overnight at 4° C with primary antibodies. Primary antibodies used include anti phospho-eIF2-α (1:1,000, #3398, Cell Signaling Technology, Beverly, MA, USA), anti-eIF2-α (1:1,000, #5324, Cell Signaling Technology), anti-ATF4 (1:1,000, ab85049, Abcam), anti-CHOP (1:1,000, #2895, Cell Signaling Technology), anti-caspase-3 (1:1000, #9662, Cell Signaling Technology), anti-cleaved-caspase-3 (1:1,000, #9664, Cell Signaling Technology), and anti-GAPDH (1:1,000, #5174, Cell Signaling Technology) antibodies. After incubation with HRP-conjugated secondary antibodies, membranes were developed with enhanced chemiluminescence (Thermo Fisher Scientific) using KODAK Image Station 4000MM PRO. Membranes were reblotted with anti-GAPDH for normalization. Densitometric analysis of immunoblots was performed using Image J software.

### Apoptosis assays

TUNEL assay was performed to detect apoptosis on cryosections of mouse eyes or in cultured HTMCs using *In Situ* Cell Death Detection TMR red kit (Roche Diagnostic) according to the manufacturer’s protocol. For HTMCs, the coverslips were fixed with 4% PFA for 1 h, followed by permeabilization for 2 min on ice in 0.1% citrate buffer containing 0.1% Triton X-100. Then the coverslips were incubated at 37° C in TUNEL reaction mix containing nucleotides and terminal deoxynucleotidyl transferase (TdT). Nuclei were stained with DAPI. Slides were visualized and photographed under a fluorescent microscope. TUNEL-positive cells were counted in ten different microscopic fields of at least three independent experiments. The index of apoptosis was presented as the percentage of TUNEL-positive cells to total (DAPI-positive).

### Detection of ROS generation in HTMCs

HTMCs seeded on a glass coverslip were transfected with si-ATF4 or si-sc for 24 h, followed by incubation with or without 100 ng/ml of tunicamycin at 37° C for 48 h. After that, HTMCs were incubated with 10 μM of 2′, 7′-dichlorodihydrofluorescein diacetate (DCFH-DA, Sigma-Aldrich) for 1 h at 37° C under 5% CO_2_. Cells were subsequently fixed and observed under an OLYMPUS FV1000 confocal microscope. Fluorescence intensity was quantified per cell in at least two hundred cells, using Image-Pro Plus software.

### Quantification of IL-8 secretion in HTMCs

IL-8 secreted into the medium was measured using the DuoSet ELISA kit for human IL-8 (R&D Systems, Minneapolis, MN, USA) according to manufacturer’s instructions. Briefly, the cell culture supernatant was collected after treatment and centrifuged to remove any visible particulate material. Standards or sample (100 μl) were added to the capture antibody pre-coated microtiter plate and incubated at room temperature for 2 h. After washing, 100 μl of detection antibody, diluted in Reagent Diluent, was dispensed into each well. The mixture was incubated at room temperature for 2 h. After washing four times, Streptavidin-HRP was added, followed by incubation for 20 min. Then substrate was added and incubated for 20 min, the plate was read immediately at 540 nm in a Thermo multiskan GO microplate reader. The IL-8 concentration was calculated according to the standard curve and normalized by protein concentration.

### Real-time RT-PCR

Total RNA of the iridocorneal tissues was isolated with the RNA extraction kit Qiagen (Duesseldorf, Germany). Total RNA was subjected to reverse transcription using a PrimeScriptTM RT Reagent kit (Takara, Dalian, China) following the manufacturer’s protocol. Real-time PCR was performed to measure the expression of ELAM-1, IL-1α, IL-1β and IL-6 at mRNA level using SYBR Premix Ex Taq (Takara, Dalian, China). The mRNA levels of target genes were normalized by GAPDH. The primer sequences are shown below.

**Table:** 

	**forward**	**reverse**
**ELAM-1**	ATGCCTCGCGCTTTCTCTC	GTAGTCCCGCTGACAGTATGC
**IL-1α**	GCACCTTACACCTACCAGAGT	AAACTTCTGCCTGACGAGCTT
**IL-1β**	GCAACTGTTCCTGAACTCAACT	ATCTTTTGGGGTCCGTCAACT
**IL-6**	TAGTCCTTCCTACCCCAATTTCC	TTGGTCCTTAGCCACTCCTTC
**GAPDH**	AGGTCGGTGTGAACGGATTTG	TGTAGACCATGTAGTTGAGGTCA

### Phagocytosis of colloidal gold by HTMCs

Briefly, 400 μl of 1% tetrachloroauric acid was dissolved in 40 ml distilled water and heated to a boil, followed by adding 800 μl of 1% citrate acid. Then the solution was refluxed and cooled down until it turned to red from blue. The finally produced colloidal gold was 25 nm in diameter. After 48 h transfection with siRNA, HTMCs were exposed to tunicamycin (100 ng/ml) for an additional 24 h, or HTMCs were infected with recombinant adenovirus for 72 h. The colloidal gold solution was added to the medium (10 μL/ml) and incubated at 37° C for 1 h. Phagocytosis of colloidal gold by HTMCs was observed under dark-field microscope (OLYMPUS).

### Statistical analysis

Values are expressed as mean ± SEM. Statistical analyses were performed using unpaired Student *t*-test when comparing two groups or ANOVA with Bonferroni’s post hoc test when comparing three or more groups. ANOVA of repeated measurements were used when comparing mouse IOP. Statistical differences were considered significant when *P*<0.05.

## Supplementary Material

Supplementary Figures
