# Supplementary material for: Activation of ATF4 triggers trabecular meshwork cell dysfunction and apoptosis in POAG
Source: Aging (Albany NY). 2021 Mar 10;13(6):8628–42. doi: 10.18632/aging.202677 (PMC8034903; doi:10.18632/aging.202677)
Supplement: Supplementary Figures [file aging-13-202677-s001.pdf]

SUPPLEMENTARY FIGURES

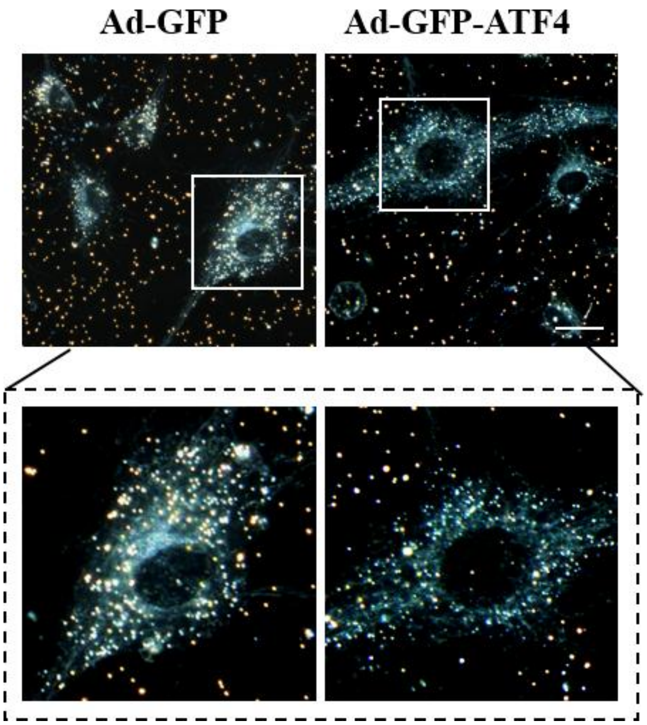

Supplementary Figure 1. Ectopic expression of ATF4 impaired the phagocytotic activity of HTMC. HTMCs were infected with Ad-GFP-ATF4 or Ad-GFP for 72 h. Phagocytosis of colloidal gold by HTMCs was examined under dark field microscope (gold, colloidal gold). Representative images were shown in upper panels (scale bar, 20 µm). Lower panels show magnified images of individual cells.

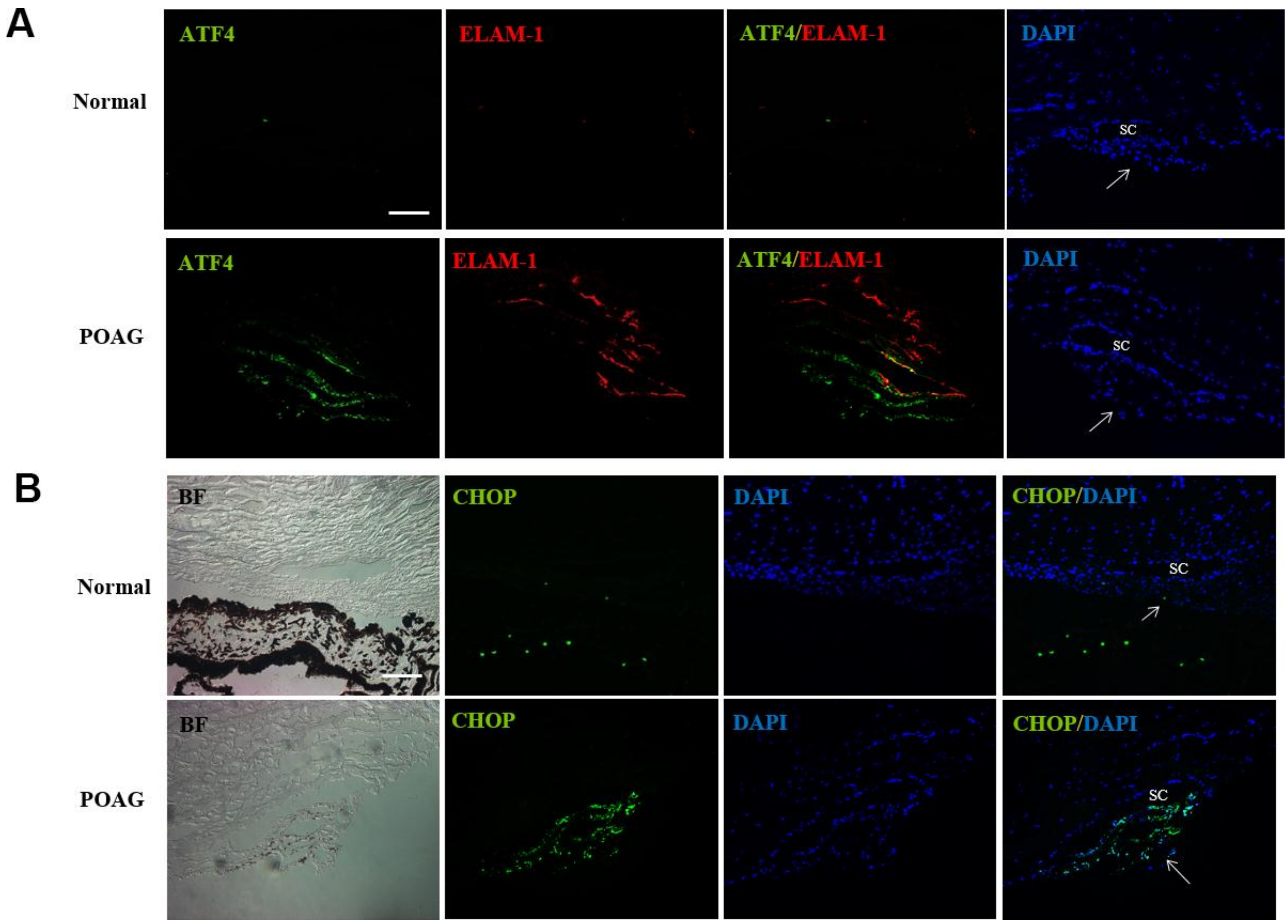

Supplementary Figure 2. Up-regulation of ATF4, ELAM-1 and CHOP in TM tissues of POAG patients. (**A**) Frozen sections of human TM tissue of POAG patients or normal controls were double stained with ATF4 (green) and ELAM-1 (red) antibodies. Partial colocalization of immunofluorescence in TM and SC in glaucomatous eyes could be seen. (**B**) Immunohistochemistry showing increased CHOP (green) expression in TM and SC of POAG patients. Arrows mark the TM. Blue: nuclear staining with DAPI. BF, bright field. SC, Schlemm's canal. Scale bar, 100μm.

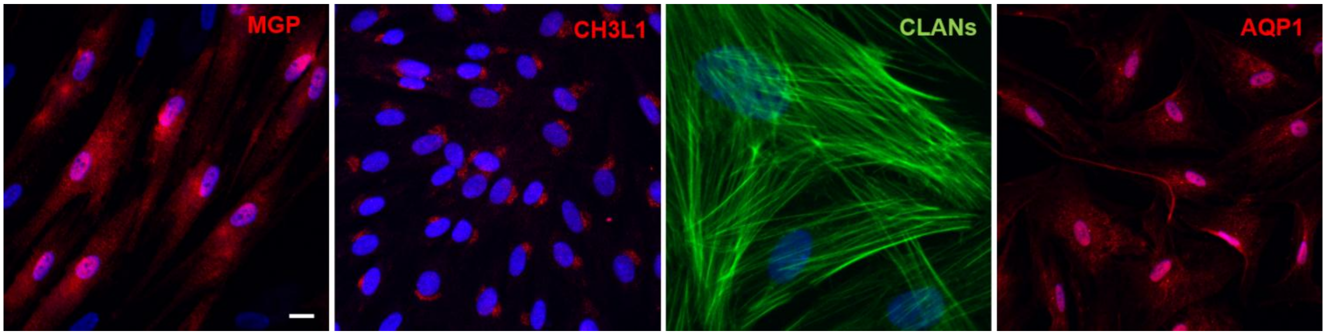

Supplementary Figure 3. Identifications of trabecular meshwork cells. Expression of MGP, CH3L1, CLANs and AQP1 was determined by immunofluorescence staining in HTMCs. blue, DAPI. Scale bar, 40μm.
